# Induction of labor versus cesarean section after 36 + 0 weeks in twin pregnancy: A review of maternal and neonatal outcomes

**DOI:** 10.1111/aogs.70271

**Published:** 2026-05-27

**Authors:** Joanna Gent, Evie Oliver, Grace Williams, Steven Lane, Kate Navaratnam, Andrew Sharp

**Affiliations:** ^1^ Harris Research Centre, Department of Women's and Children's Health University of Liverpool UK; ^2^ Liverpool Women's Hospital Liverpool UK; ^3^ London North West Healthcare Trust, London UK; ^4^ Department of Health Data Science Institute of Population Heath, University of Liverpool UK

**Keywords:** induction of labor, multiple pregnancy, twins

## Abstract

**Introduction:**

Our aim was to assess maternal and perinatal morbidity associated with induction of labor (IOL) and elective cesarean section (ECS) in uncomplicated twin pregnancies delivered >36 weeks' gestation.

**Material and Methods:**

A retrospective review of all twin pregnancies, irrespective of chorionicity, that underwent ECS and IOL at the Liverpool Women's Hospital (LWH) over a 10‐year period.

**Results:**

Three hundred and eighty‐two women underwent IOL and 326 had ECS between January 2010 and December 2020 at LWH. 71.2% (*n* = 272) achieved vaginal delivery of both babies in the IOL group, 4.2% (*n* = 16) required CS for the second twin. There was no difference in blood loss >1500 mLs (7.4% vs. 6.8%, RR 0.92 (0.54–1.58), *p* = 0.77). Admission of one or both twins to the neonatal unit was higher following IOL than ECS (13.5% vs. 10%, RR 1.35 (1.01–1.81), *p* = 0.04 and 5.1% vs. 2.9%, RR 1.75 (1.03–2.97), *p* = 0.04, respectively). There was no impact of chorionicity on outcomes.

**Conclusions:**

Existing consensus on mode of delivery of twins at term suggests both IOL and ECS are safe options. We found an association between increased neonatal admissions following IOL compared to ECS, which has also been observed in line with previous epidemiological studies. Our study broadens our knowledge and strengthens the case that this should be discussed when counseling women about intended mode of birth.

## Graphical abstract

Women with a twin pregnancy who undergo induction of labor or elective cesarean section at term are comparable for blood loss but with higher admissions to the neonatal unit.

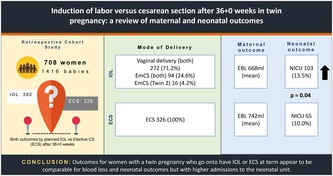

**Abbreviations**
CS: Cesarean section
EBL: Estimated blood loss
IOL: Induction of labor
LWH: Liverpool Women's Hospital
NNU: Neonatal unit
TBS: Twin birth study

**Key message**

Outcomes for women with a twin pregnancy who go onto have Induction of Labor or Elective Cesarean Section at term appear to be comparable for blood loss and neonatal outcomes but with higher admissions to the neonatal unit.

## 1 INTRODUCTION

Multiple pregnancies account for approximately 1.5% of the pregnant population.^1^ They are associated with significant perinatal morbidity and mortality, with the gap between singleton and twin stillbirth and neonatal mortality rates widening despite much focus and intervention in recent years.^2^ Prematurity is the leading contributing factor, with over 50% of multiple pregnancies delivered prior to 37 weeks; however, for those that do reach term, there is ongoing consideration as to the optimal timing and method of delivery.

In general, increased gestational age at delivery is associated with decreased perinatal and neonatal mortality.^2^ However, there is a simultaneous rise in the risk of stillbirth with advancing gestation in twins, from 3.4 per 1000 at 37 weeks to 10.6 per 1000 at 38 weeks in dichorionic (DC) pregnancies and 4.5 per 1000 at 36 weeks to 9.6 per 1000 at 37 weeks in monochorionic (MC) pregnancies.^3^ The balance of these factors is reflected in current national and international guidance which advises offering “planned birth” from 37^+0^ weeks for DC twins and 36^+0^ weeks for uncomplicated monochorionic diamniotic (MCDA) twins.^4^, ^5^, ^6^, ^7^

Both elective cesarean section and planned vaginal delivery are considered “safe choices” in uncomplicated twin pregnancy, where the leading twin is cephalic and in the absence of significant size discordance.^4^ However, a paucity of evidence exists for the preferential mode of delivery of twin pregnancies at term. The majority of guidance extrapolates findings from the Twin Birth Study (TBS), a randomized controlled trial comparing planned vaginal delivery and cesarean section (CS) at 32 + 0 to 38 + 6 weeks.^8^ The study concluded that neither delivery method significantly influenced serious neonatal morbidity or fetal/neonatal death.^8^ However, when counseling about mode of delivery in the clinic we are in reality discussing what the likelihood of success would be should the planned method of delivery be initiated, rather than what occurred in the TBS which was to randomize to a method of delivery which a proportion of women, for a variety of reasons, did not then undergo. In the TBS, almost half of deliveries in both the planned cesarean and planned vaginal delivery groups were preterm, a major determinant of neonatal outcome and a likely significant factor in masking any potential benefit of CS at term, something previously observed in epidemiological studies and subsequent secondary analyses of the TBS.^8^, ^9^, ^10^ When counseling women in the antenatal clinic we should be counseling them about their chance of delivering according to the planned mode of delivery, as per our study rather than as per the TBS where many did not deliver according to their intended, randomized, mode of delivery. For an RCT, this analysis via intention to treat is highly valuable, but for the clinician counseling a woman about mode of delivery it is the outcomes from the actual mode of delivery intended that she will be interested in.

The decision on how to deliver a term twin pregnancy is influenced by many factors; however, one of the foremost is the outcomes for the neonates, ensuring a safe delivery of both fetuses with limited complications. These complex decisions should be informed, utilizing data on the risks of each method of delivery as well as other obstetric factors, such as parity, previous mode of birth, and BMI.

The aim of this study was to assess maternal and neonatal morbidity associated with induction of labor (IOL) and elective cesarean section (ECS) in uncomplicated twin pregnancies delivered ≥36 + 0 weeks' gestation.

## 2 MATERIAL AND METHODS

This project was a retrospective cohort study of twin pregnancies that delivered at Liverpool Women's Hospital (LWH), a tertiary maternity center in the UK, between January 2010 and December 2020. All women that gave birth to twins between 36^+0^ and 40^+6^ weeks gestation during the 10‐year period were included in the study. Data was collected on onset of labor; induction of labor (IOL) or elective cesarean section (ECS) and mode of delivery, vaginal or CS. IOL methods included artificial rupture of membranes (ARM), Prostaglandin (PGE2), and Propess pessary® (PGE2) followed by augmentation with Syntocinon (SYN). Data on emergency cesarean section (EMCS) for failed IOL or other indications, such as fetal distress, was also recorded.

In addition, baseline maternal and neonatal data were collected using electronic and paper patient records including maternal demographics, indication for delivery, bishops score, and twin position. Maternal outcomes of interest included intended and actual delivery method, incidence of operative deliveries, and estimated blood loss (EBL). Neonatal morbidity was assessed using APGAR scores five minutes after delivery, cord pH, and admission to the neonatal unit (NNU).

### 2.1 Statistical analysis

Data was firstly summarized using descriptive statistics, for example, mean, median, and corresponding measures of variability for continuous data and counts and percentages for categorical data. Relative Risks and adjusted odds ratios using logistic regression models were calculated to assess the relationship between mode of delivery (IOL v CS) and maternal/neonatal outcomes.

The initial list of variables considered for the multivariate regression model was BMI, Smoker, Maternal Age, Ethnicity, Fertility treatment, Gestational Age and Parity. A univariate analysis of these variables only identified Maternal Age and Smokers as having a statistically significant relationship with the outcome (*p* < 0.05). Consequently, only these two variables were carried forward into the multivariate model.

## 3 RESULTS

Seven hundred and eight women delivered twins following IOL or ECS between January 2010 and December 2020 at LWH. With the exception of BMI and smoking, there were no differences between the induced and CS cohorts. This is likely a reflection of normal variation in a non‐randomized population (Table 1). Data are presented as mean + − SD, *n* (%) and median (lower quartile–upper quartile).

**TABLE 1:** Demographic data and baseline characteristics.

	IOL(*n* = 382)	Elective CS(*n* = 326)	Relative risk (95%CI) or *p* value
Age	31.5 ± 5.6	32.1 ± 5.5	NS
BMI	26.0 ± 5.6	26.9 ± 5.7	*p* = 0.03
Ethnicity			
White Other	328 (85.9) 54 (14.1)	278 (85.3) 48 (14.7)	NS NS
Fertility treatment	96 (25.1)	92 (28.2)	NS
Smoker	14 (3.7)	23 (7.1)	P = 0.04
DCDA	305 (79.8)	272 (83.4)	NS
MCDA	77 (20.2)	54 (16.6)	NS
Parity			
0 1 2 ≥3	162 (42.4) 125 (32.7) 60 (15.7) 35 (9.2)	132 (40.5) 117 (35.9) 44 (13.5) 33 (10.1)	NS
Gestational age (weeks) Gestational age (days)	37.2 260.3 ± 5.1	37.2 260.2 ± 4.2	NS
Indication for delivery			
Gestational age Maternal factors Diabetes Placenta Praevia Hypertension Cholestasis APH PPROM	327 (85.6) 4 (1.0) ‐ 13 (3.4) 11 (2.9) 1 (0.3) 13 (3.4)	311 (95.4) 2 (0.6) 4 (1.2) 2 (0.6) ‐ ‐ ‐	
Fetal factors			
sFGR Fetal abnormality RFM	9 (2.4) 1 (0.3) 3 (0.8)	3. (0.9) 4 (1.2) ‐	
Bishop score			
0 1 2 3 4 5 ≥6 Not known	20 (5.2) 30 (7.9) 48 (12.6) 54 (14.1) 63 (16.5) 43 (11.3) 99 (25.9) 25 (6.5)		
Presentation twin 2			
Cephalic Breech Transverse Compound Not known	220 (57.6) 126 (33.0) 16 (4.2) 6 (1.6) 14 (3.7)		

54% (*n* = 382) of women underwent IOL, and 46% of women (*n* = 326) had an ECS. Delivery outcomes are summarized in Table 2. 75.4% (*n* = 288) of women who were induced achieved a vaginal delivery of either one or both twins. 71.2% (*n* = 272) achieved a vaginal delivery of both twins, 4.2% delivered Twin 1 vaginally and then Twin 2 by CS, and 26.4% delivered both babies by CS. No women who underwent IOL had a previous cesarean section.

**TABLE 2:** Delivery outcomes of twin pregnancies.

	IOL(*n* = 382)	Elective CS(*n* = 326)	Relative risk (95%CI) or *p* value
Mode of delivery			
Elective C/S Vaginal delivery (both) Emergency CS (both) Emergency C/S (Twin 2)	‐ 272 (71.2) 94 (24.6) 16 (4.2)	326 (100) ‐ ‐ ‐	
Vaginal deliveries			
Instrumental both Instrumental T1 Instrumental T2	32 (8.4) 24 (6.3) 23 (6.0)	‐ ‐ ‐	
Significant tears			
OASIS 3rd/4th degree	1 (0.3)	‐	
Estimated blood loss (mean)	668.2	742.3	*p* = 0.02
0‐499 mls 500–999 mls 1000‐1499 mls >1500 mls	156 (40.8) 146 (38.2) 54 (14.1) 26 (6.8)	29 (8.9) 227 (69.6) 46 (14.1) 24 (7.4)	4.59 (3.18–6.63) *p* < 0.0001 0.64 (0.56–0.74) *p* < 0.0001 1.01 (0.70–1.44) *p* = 0.99 0.92 (0.54–1.58) *p* = 0.77

27.4% (*n* = 79) of women who delivered vaginally had an instrumental delivery. 30.4% (*n* = 24) for T1, 29.1% (*n* = 23) for T2, and 40.5% (*n* = 32) for both twins. Most women who experienced an instrumental delivery had an episiotomy (96%) and 1 woman had an obstetric anal sphincter injury (OASIS) 3rd/4th degree tear. 3 women had an episiotomy for T1 and later required an EMCS for T2.

In those women who started IOL but ended up with a CS for both babies 20 from 94 were MCDA (21.2%). Of these, 9 from 20 (45%) underwent CS due to fetal distress which is higher than the 17 from 74 for DCDA twins (23%). However, we cannot give any more detail on the cause of the fetal distress and on such low numbers we would be cautious to attribute the fetal distress to intrapartum TTTS. For those with twin, 1 delivered vaginally and twin 2 by CS only 2 from 16 were MCDA (12.5%). No babies had a postnatal diagnosis of TTTS.

The mean EBL for women having IOL was 668.2 mL and for those who had an ECS was 742.3 mL. Moderate blood loss between 500 to 999 mL was recorded for 69.9% (*n* = 226) of women who had an ECS compared to only 38.2% (*n* = 146) from IOL. Similar numbers of women had blood loss >1500 mL across both delivery methods: 6.8% (*n* = 26) during IOL and 7.4% (*n* = 24) in ECS. There was no difference in blood loss >1500 mLs between delivery methods (7.4% vs. 6.8%, RR 0.92 (0.54–1.58), *p* = 0.77).

One thousand four hundred and sixteen babies were born to the 708 mothers in the study. All deliveries resulted in live births of both babies (Table 3). APGAR scores <7 were recorded for 34 babies, which is considered an indicator of poor neonatal health.^11^ Of these babies, 79.5% (*n* = 27) were delivered from women who underwent IOL, whereas only 26.5% (*n* = 9) were delivered by ECS.

**TABLE 3:** Neonatal outcomes of twin deliveries.

	IOL(*n* = 764)	Elective CS(*n* = 652)	aOR not relative risk (95%CI) or *p* value
Livebirth	764 (100)	652 (100)	‐
APGAR <7 at 5 mins			
Twin 1 Twin 2 Not recorded	9 (1.2) 16 (2.1) 5 (0.7)	3 (0.5) 6 (0.9) 4 (0.6)	12.8 (0.73–223.39) *p* = 0.08 2.28 (0.90–5.75) *p* = 0.08 ‐
Arterial cord pH <7.10			
Twin 1 Twin 2 Not recorded	7 (0.9) 34 (4.5) 177 (23.2)	0 0 483 (74.0)	
NNU admission			
Total Twin 1 only Twin 2 only Both twins Not Known	103 (13.5) 25 (3.3) 39 (5.1) 39 (5.1) 0 (0)	65 (10.0) 24 (3.7) 22 (3.4) 19 (2.9) 1 (0.2)	1.35 (1.01–1.81) *p* = 0.04 0.88 (0.52–1.53) *p* = 0.67 1.51 (0.91–2.50) *p* = 0.11 1.75 (1.03–2.97) *p* = 0.04

11.9% (*n* = 168) of twins delivered at LWH required admission to the Neonatal Unit (NNU). More babies who underwent IOL were admitted to the NNU; 13.5% (*n* = 103), compared to 10.0% (*n* = 65) of babies who were delivered by ECS. Following IOL, 3.3% of Twin 1 and 5.1% of Twin 2 were admitted to NNU. Twins delivered by ECS had similar NNU admission rates (3.7% T1 (*n* = 24), and 3.4% for T2 (*n* = 22)). Neonatal admission for both twins was higher following IOL (*p* = 0.04 and 5.1% vs. 2.9% RR 1.75 (1.03–2.97), *p* = 0.04, respectively).

We performed a logistic regression analysis on the two significant predictors (BMI and smoking) of CS over IOL from Table 1. The analysis confirms that being a smoker and having a higher BMI increase the chance of choosing CS compared to IOL (Table 4). However, when combined, both have borderline significance.

**TABLE 4:** CS vs. IOL logistic regression. Models 1 and 2 are univariate models and model 3 is the multivariate model combining both significant variables.

Model 1	Beta	St error	OR	95% CI	Statistical significance
Smoker	0.691	0.348	1.995	(1.009, 3.945)	*p* = 0.047
Model 2					
BMI	0.027	0.013	1.028	(1.001, 1.055)	*p* = 0.042
Model 3					
Smoker	0.638	0.350	1.893	(0.954, 3.757)	*p* = 0.068
BMI	0.026	0.014	1.026	(0.999, 1.054)	*p* = 0.058

## 4 DISCUSSION

From our retrospective cohort study, we found the most frequent outcome from IOL to be vaginal delivery of both twins, experienced by 71.2% of participants. Similar success rates have been reported in other studies.^12^, ^13^ However, the TBS concluded lower rates of successful IOL, with only 52.6% of women assigned VD achieving this outcome. This could be attributed to including twins from 32 weeks' gestation who are more likely to need an EMCS due to prematurity, reducing the chance of successful labor or intrapartum fetal concerns. One study described 79% successful delivery after IOL, which is higher than both our study and the TBS.^9^ Another cohort excluded women with previous c‐sections and achieved an 80% vaginal delivery rate and 20% CS rate, and as such may be reflective of our own study where no women induced had a previous CS.^14^ We are therefore unable to comment on the effectiveness of induction of labor in women with twins and a previous CS scar.

We demonstrated an incidence of 4.18% for birth of Twin 1 vaginally and Twin 2 by EMCS, which is similar to the TBS, with 4.2% in their IOL cohort. The similar rate is likely because there is a low threshold to deliver twins by EMCS when there are signs of fetal compromise.^15^

Our study concluded that around 25% of those women who achieved a vaginal delivery required instrumental assistance in the delivery of their twins. Intrapartum intervention with forceps or ventouse is not widely explored in the literature for twins compared to singletons and is not reported in the TBS.^9^ While we acknowledge that there are cultural and institutional variations in instrumental delivery rates, it is likely that our findings would be replicated in other similar healthcare settings.

This study has demonstrated that while the average blood loss was similar for both IOL and ECS, more women experienced moderate blood loss (500–999 mL) when delivered by ECS. Guidance on twin birth supports the need for active planning to minimize blood loss in multiple pregnancy.^4^ We found blood loss >1500 mL to be similar between delivery methods, as others have also found,^9^ helping to confirm that expected blood loss and risk of postpartum hemorrhage (PPH) should not be a factor when counseling about mode of delivery in twin pregnancy. While we accept that no adjustment for multiple analyses was undertaken, the mean values before adjustment remain statistically significant at *p* < 0.05.

However, it is important to consider that of the women in our sample who underwent IOL, around 28% required an EMCS. It is well documented that EMCS results in greater blood loss than ECS^16^ and therefore average EBL for IOL participants includes both women who had an uncomplicated vaginal delivery and those who had an EMCS, a limitation also acknowledged in the TBS.

All methods of delivery in our study resulted in the live birth of both babies as was also found in the TBS,^17^ reiterating the overall safety of both IOL and ECS to deliver twins at, or near term. In our study, more babies delivered following IOL had low APGAR scores 5 min after delivery than babies born by ECS. This finding is likely to be multifactorial, including factors such as longer delivery and inter‐twin delivery times and acute intrapartum factors leading to fetal distress. Although trends are similar across delivery methods, the TBS had fewer twins with low APGAR scores than our project, although this is likely due to the much lower criteria for classification of low APGAR score (<4). The inclusion of premature twins in the planned vaginal delivery arm of the TBS may have resulted in the higher EMCS rate than we found in our term cohort. C‐sections have been suggested by a recent meta‐analysis to have a protective effect on APGAR scores and early neonatal warning signs,^18^ which may provide further reason for this correlation. In contrast to this, another leading study found no difference between IOL and ECS in neonatal APGAR scores. This study is more directly comparable to ours; similarly, it includes APGAR <7, 5 mins post‐delivery. Their findings emphasized that twins delivered by ECS were more likely to require CPAP than those delivered by IOL.^13^ This indicates that the method of delivery and the gestation at birth may have a greater influence on the treatment required. Caution should be exerted due to multiple analyses undertaken for neonatal admission but the mean values before adjustment remain statistically significant at *p* < 0.05.

In our study, more babies delivered by IOL required NNU admission although we did not assess the reason for admission or length of stay on the NNU. It is likely that the indication for admission is minor as the TBS found no difference in the primary outcome between delivery methods despite being a cohort which included neonates born at an earlier gestation who would be expected to be at greater risk of neonatal morbidity. Twins delivered by ECS had similar NNU admission rates irrespective of birth order; however, following IOL, more second Twins were admitted. Similar findings were documented in a systematic review and meta‐analysis investigating neonatal outcomes of twins comparing birth order to mode of delivery.^19^ While it was concluded that Twin 1 has lower neonatal morbidity following a vaginal delivery, the TBS described worse outcomes for Twin 2 irrespective of delivery method. This suggests neonatal outcomes are dependent on a combination of factors including birth order, gestation, and mode of delivery, and it should be borne in mind that while outcomes from IOL in our cohort reflect the final mode of birth, namely vaginal, instrumental, and emergency CS, not just the decision on planned initiation of induction.

Our outcomes are broadly in line with the secondary analysis of the TBS which looked at >36 + 0 weeks births,^9^ and taken together with our study we are able to aid counseling on the risks and benefits of planned birth mode for “full term” twins and the impact on neonatal and maternal outcomes. This was with the aim to help inform obstetricians and guide families when choosing delivery methods. The full scope of this study is limited by the lack of long‐term follow‐up.

This study is limited by its retrospective nature and the potential for confounding as a consequence of this design. We used a cut‐off approach to determine the significance of variables to be included in the multivariate model with a p value of <0.005 considered significant. We decided not to adjust the p value for multiple testing; therefore, statistically significant results should be treated with caution due to the increased risk of false results.

## 5 CONCLUSION

This study has concluded that both IOL and ECS are safe methods of delivery for twins at term. Most women who choose to have a vaginal delivery achieve this; however, around 25% of women will require obstetric assistance via an instrumental delivery for one or both twins. Of those who are not able to safely progress to a vaginal delivery, it is most likely that both twins will be delivered by EMCS. Average blood loss is similar for both vaginal and cesarean deliveries, and there was also no difference in blood loss >1500 mLs between delivery methods. Overall, maternal outcomes in our cohort are in line with those within the literature. There appeared to be no difference in outcomes related to chorionicity.

All twins in our cohort were live births. A very small number of babies overall had low APGAR scores, more so in twins delivered vaginally. More babies born following IOL were treated in the NNU compared to babies born by ECS.

We anticipate that this study will aid decision making for women and clinicians when choosing a planned mode of delivery at term in twin pregnancy. It should be used in association with the TBS, which provides additional information on the chance of achieving the planned delivery method. However, despite our evidence of a higher rate of admission to the NNU, our study is too small to address safety for rare perinatal outcomes and, as such, we suggest that further large‐scale studies on twin pregnancy with a detailed assessment of neonatal outcomes are required.

## AUTHOR CONTRIBUTIONS

JG conceived the study and performed initial analysis of data and wrote the initial manuscript, EO performed the data collection, KN provided oversight and review, GW revised the manuscript for submission, SL provided statistical expertise, AS reviewed and edited the manuscript.

## CONFLICT OF INTEREST STATEMENT

The authors declare that they have no known competing financial interests or personal relationships that could have appeared to influence the work reported in this paper.

## ETHICS STATEMENT

Ethical approval was not required due to the retrospective study design; however, data was anonymized and analyzed on secure trust devices to ensure confidentiality. This project received approval by LWH audit department (2020/039).

## Data Availability

The data that support the findings of this study are available on request from the corresponding author. The data are not publicly available due to privacy or ethical restrictions.
